# Supplementary material for: A CRISPR–Cas9-triggered strand displacement amplification method for ultrasensitive DNA detection
Source: Nat Commun. 2018 Nov 27;9:5012. doi: 10.1038/s41467-018-07324-5 (PMC6258682; doi:10.1038/s41467-018-07324-5)
Supplement: Supplementary file 1 — Supplementary Information [file 41467_2018_7324_MOESM1_ESM.pdf]

Supplementary Information

A CRISPR-Cas9-triggered strand displacement amplification method for ultrasensitive DNA detection

Zhou *et al.*

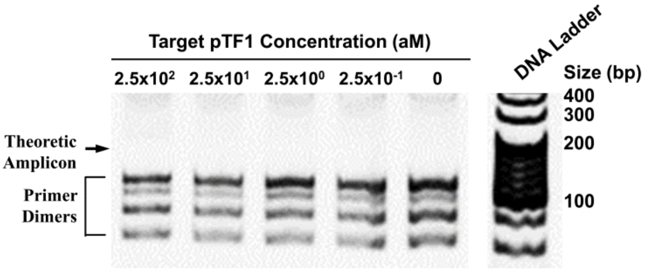

Supplementary Figure 1. Representative PAGE analysis showing CRISDA cannot initiate amplification of target pTF1 without Cas9. The representative gel analysis illustrating CRISDA fails to produce any amplicon from attomolar target pTF1 in the absence of the Cas9 protein. The arrow indicates the position of theoretic 186 bp amplicon.

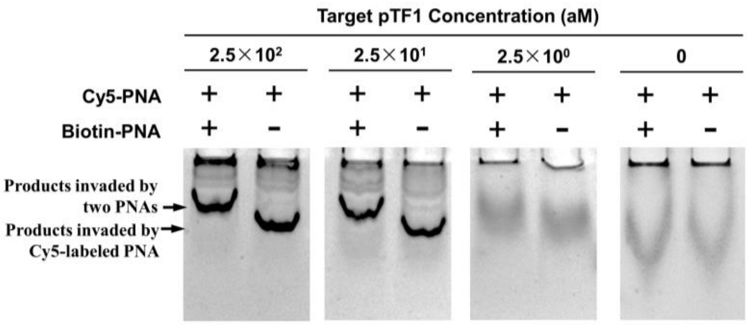

Supplementary Figure 2. Representative PAGE analysis showing that CRISDA detects attomolar pTF1 targets in combination with the PNA. The CRISDA reactions proceed at 37 °C for 90 min and the arrows indicate that PNA-invasion is confirmed by altered migration of Cy5-positive species in 6% native PAGE. Although lanes without target pTF1 also reveal Cy5-positive species, they do not interact with Biotin-labeled PNA and thus are not pulled down and interfere with subsequent fluorescence measurements.

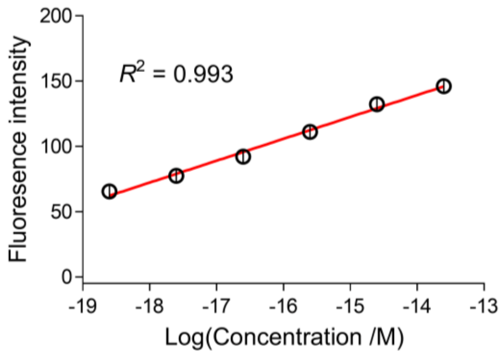

Supplementary Figure 3. Representative calibration curve for target pTF1 detection with CRISDA. Cy5 fluorescence intensities from CRISDA combined with PNA invasion-mediated fluorescence measurements are plotted against log of concentration of target pTF1, showing significant correlation between the target concentration and detected fluorescence intensity. The red line is the linear regression fit (R^2 = 0.993).

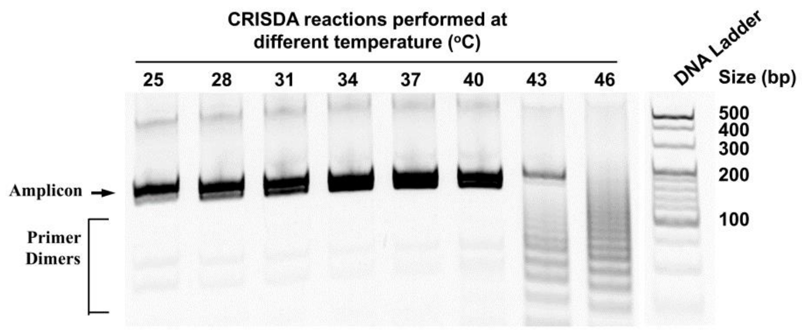

Supplementary Figure 4. Representative PAGE analysis showing the great temperature tolerance of CRISDA. CRISDA reactions amplifying 250 aM of target pTF1 are conducted at temperature ranging from 25 to 46 °C. Successful amplifications are obtained when CRISDA reactions take place between 28 and 43 °C, indicating large temperature tolerance of CRISDA.

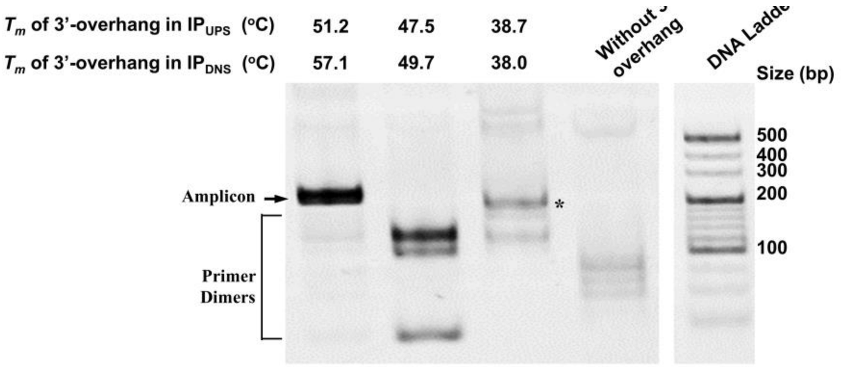

Supplementary Figure 5. Representative PAGE analysis showing the critical function of the 3' overhang in IP primers. The melting temperature of 3' overhangs must be over 50 °C for successful CRISDA reactions (*: Sequencing verification confirms that it is a product from the primer dimer rather than the targeted amplicon.) CRISDA reactions are carried out at 37 °C for 90 min with 250 aM pTF1 as the target.

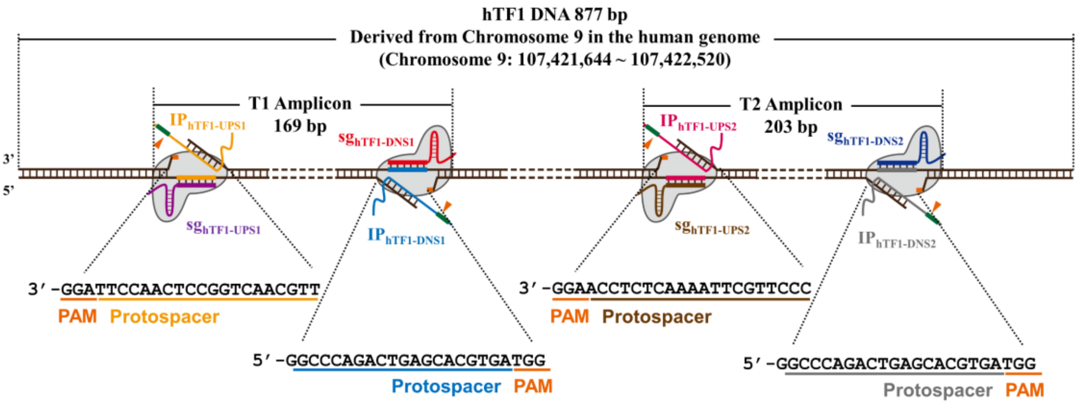

Supplementary Figure 6. Schematic of CRISDA-based DNA amplification and detection towards an 877 bp DNA fragment hTF1. DNA fragment hTF1 is derived from Chromosome 9 in the human genome. Two pairs of sgRNAs ($sg_{hTF1\text{-}UPS1/DNS1}$, and $sg_{hTF1\text{-}UPS2/DNS2}$) and IP primers ($IP_{hTF1\text{-}UPS1/DNS1}$, and $IP_{hTF1\text{-}UPS2/DNS2}$) are designed to specifically target and amplify a 169 bp (T1) and 203 bp (T2) region in hTF1, respectively. In genomic CRISDA, the same sgRNAs and IP primer pairs are used to amplify the corresponding T1 and T2 regions in the human genomic DNA extracted from HEK293 cells.

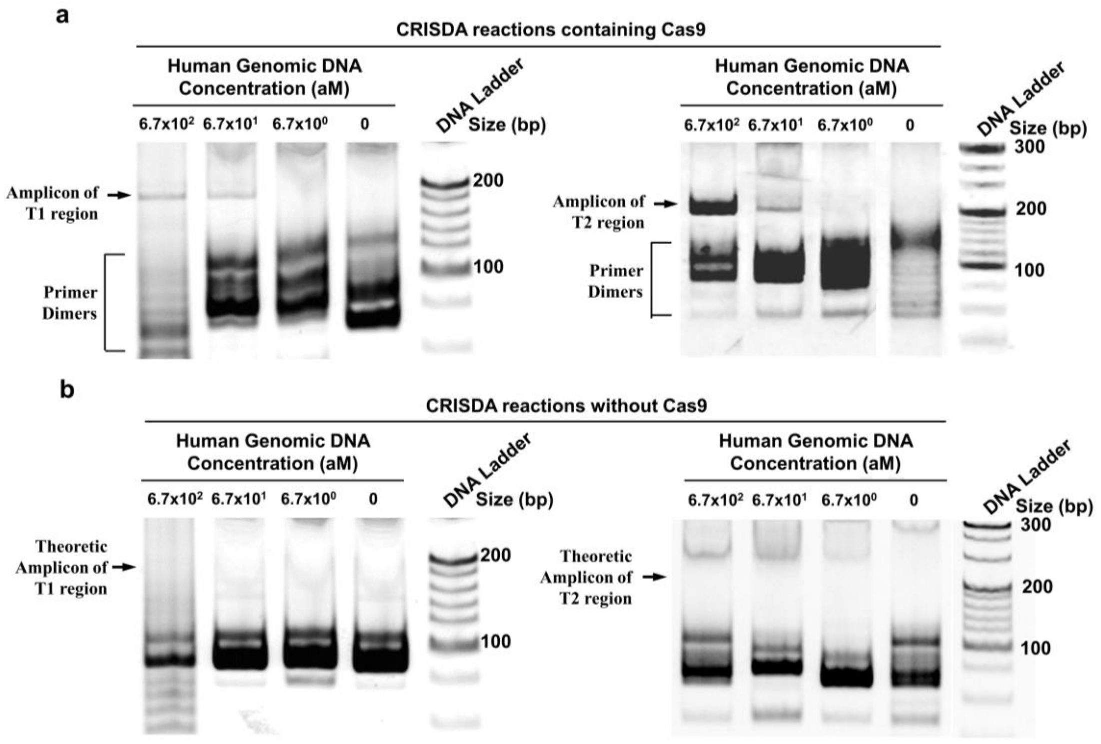

Supplementary Figure 7. PAGE analyses showing Cas9 protein is critical in genomic CRISDA amplification reactions. (**a**) CRISDA reactions containing Cas9 successfully amplify T1 (the left graph) and T2 (the right graph) from 67 aM human genomic DNA extracted from HEK293 cells. The arrows indicate successful amplification of the targeted amplicons. (**b**) CRISDA reactions fail to produce any amplicon from human genomic DNA in the absence of Cas9 protein. The arrows indicate the position of theoretic amplicon which is absent from the CRISDA reactions without Cas9.

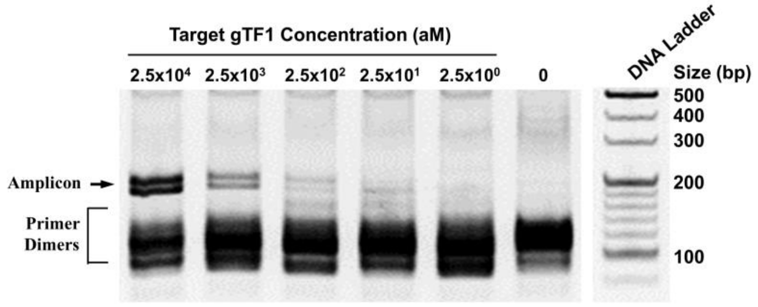

Supplementary Figure 8. PAGE analysis showing CRISDA is capable of highly sensitive amplification of GMO fragments gTF1. gTF1 is PCR amplified from the genome of a genetically-modified soybean, MON87705. The arrow indicates successful amplification of the targeted amplicon.

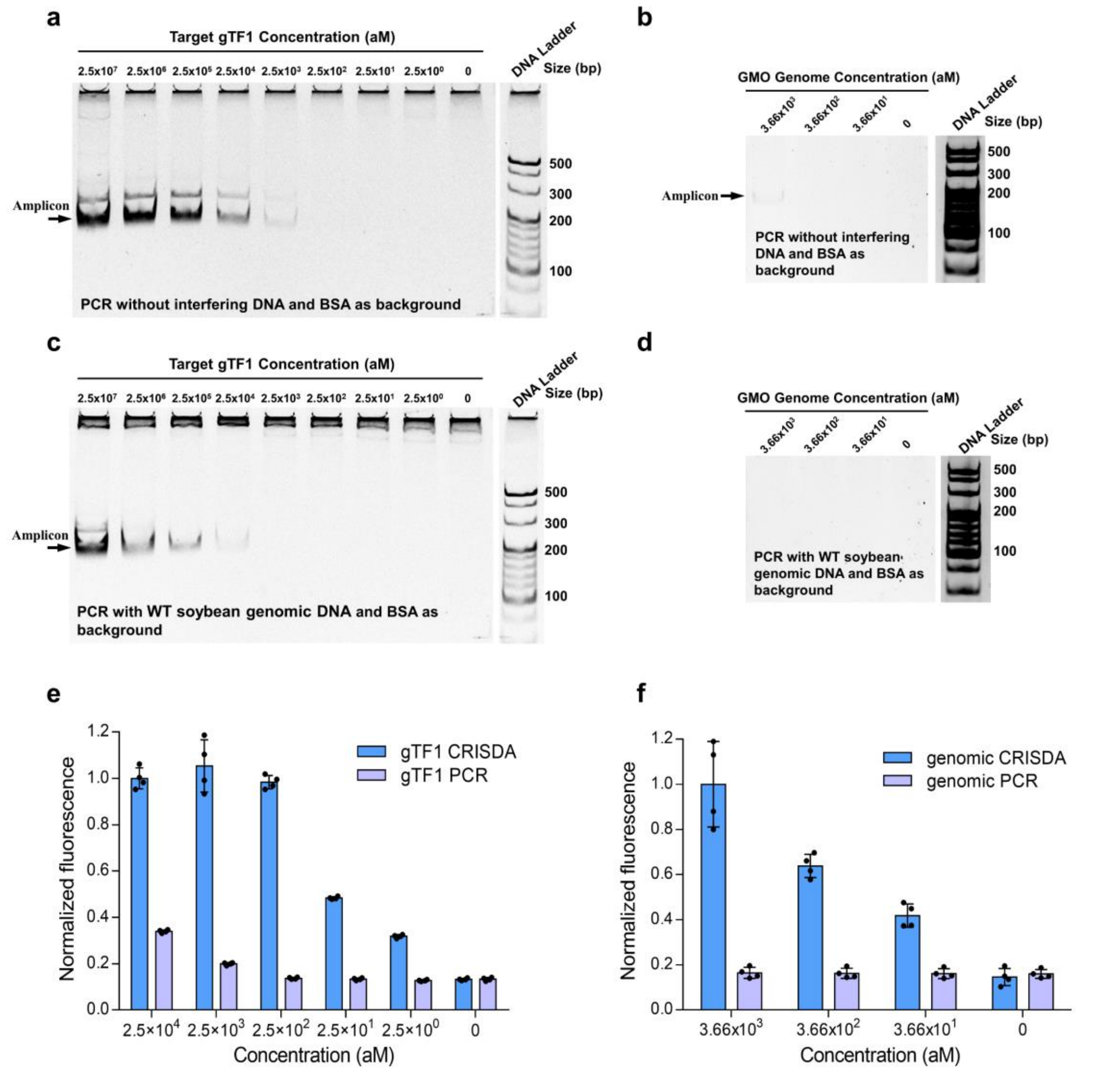

Supplementary Figure 9. PAGE analyses and PNA invasion-mediated endpoint measurements towards products amplified by traditional PCR. PAGE analyses reveal that PCR successfully amplifies (**a**) 1 ng (25 pM) to 0.1pg (2.5 fM) of target gTF1 and (**b**) 50 ng (3.66 fM) GMO genomic DNA diluted without background. In the presence of interfering DNA and BSA as background, PCR fails to produce detectable amplicons (**c**) below 25 fM gTF1 and (**d**) 3.66 fM GMO genomic DNA as templates. (**e**) Weak fluorescent signals are observed by the PNA invasion-mediated method from the PCR products containing 25 and 2.5 fM GMO fragment gTF1 as templates. (**f**) No fluorescence variations are observed from the PCR products using GMO genomic DNA as templates. (Fluorescence signals of CRISDA products are adopted from Fig. 4b and 4c) $n = 4$ technical replicates, two-tailed Student's t test, bars represent mean ± s.d.

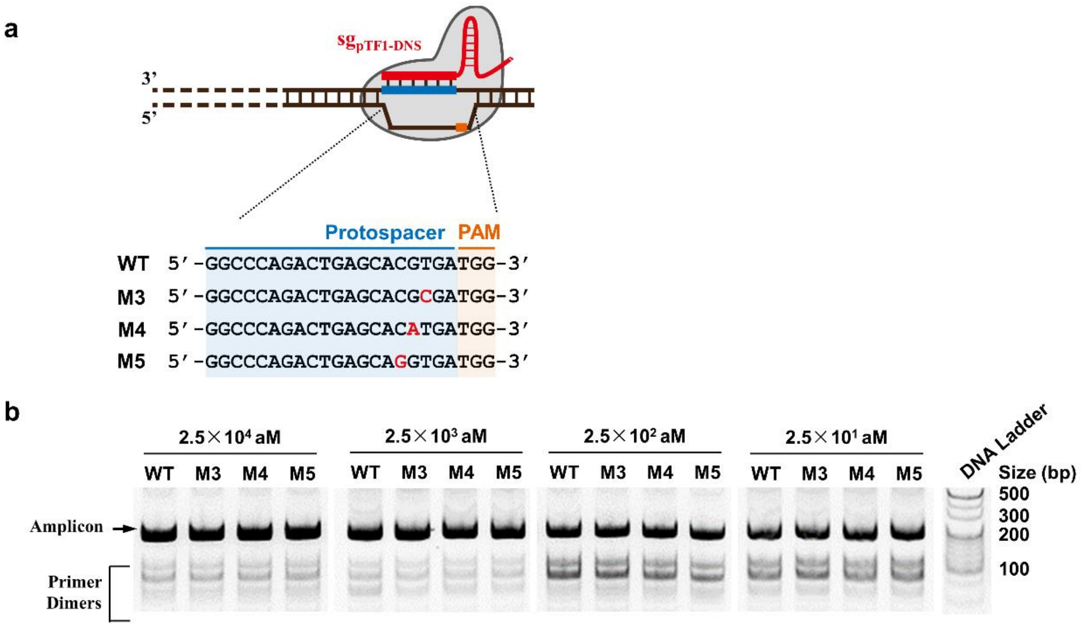

Supplementary Figure 10. CRISDA cannot discriminate single-nucleotide mutations at the +3 ~ +5 sites in the seed sequence. (**a**) Schematic of wild type pTF1 fragment and various mutants bearing single-nucleotide mutations at the +3 ~ +5 sites in the seed sequences. WT: wild type, M3: +3 mutant, M4: +4 mutant and M5: +5 mutant. (**b**) PAGE analyses showing that CRISDA cannot discriminate pTF1 fragments with single-nucleotide mutations at the +3 ~ +5 sites in the seed sequence.

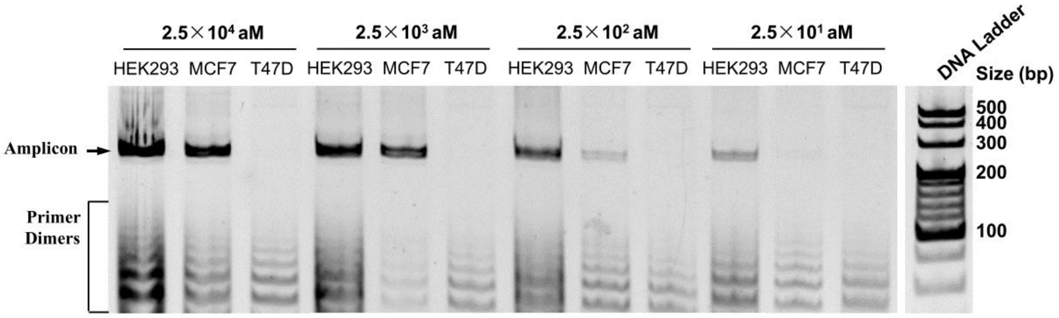

Supplementary Figure 11. PAGE analyses showing that CRISDA discriminates hTF2 fragments with single-nucleotide mutations at the rs3803662 site.

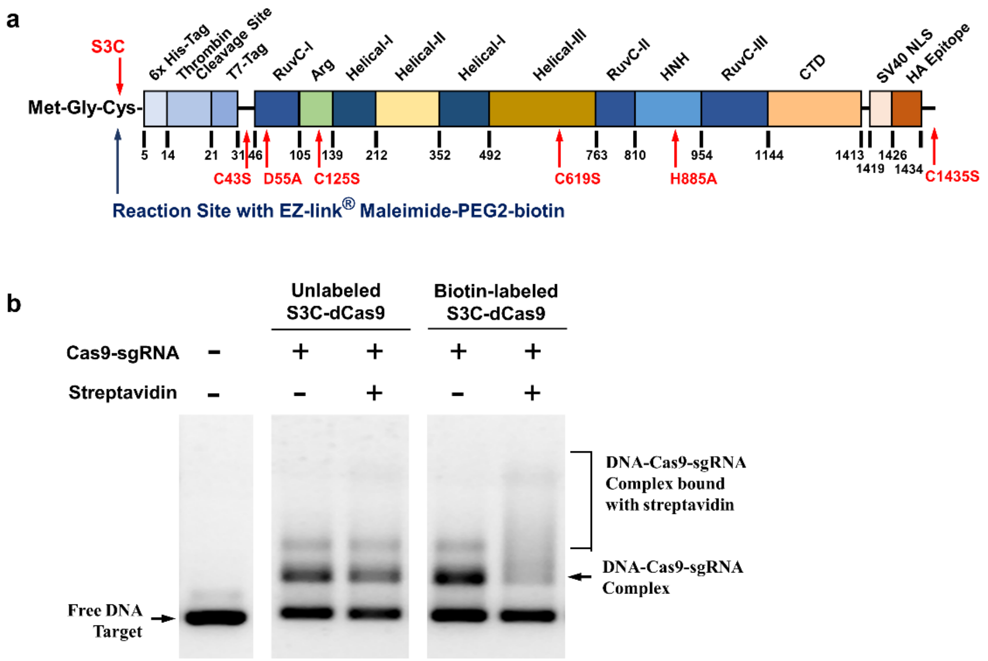

Supplementary Figure 12. Site-specific biotin labeling of S3C-dCas9. (**a**) To construct a dCas9 protein with single cysteine at the N-terminus, the 3rd Serine in Cas9 from pET-28a/Cas9-Cys plasmid (Addgene: Plasmid #53261) is mutated to Cysteine (S3C) and other Cysteines are mutated to Serine (C43S, C125S, C619S, and C1435S). S3C mutation provides a single reaction site for the EZ-link® Maleimide-PEG2-biotin. dCas9 mutations are also indicated in the domain schematic. (**b**) Agarose gel electrophoresis revealing successful biotinylation of active S3C-dCas9.

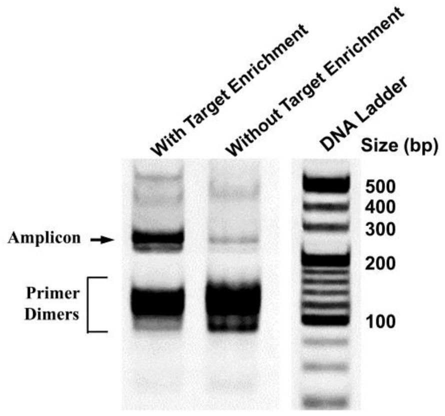

Supplementary Figure 13. Cas9-mediated enrichment of target DNA enhances sensitivity and reliability of CRISDA. The arrow indicates successful amplification of the targeted amplicon. (reactions performed at the hTF1 concentration of 2.5 aM).

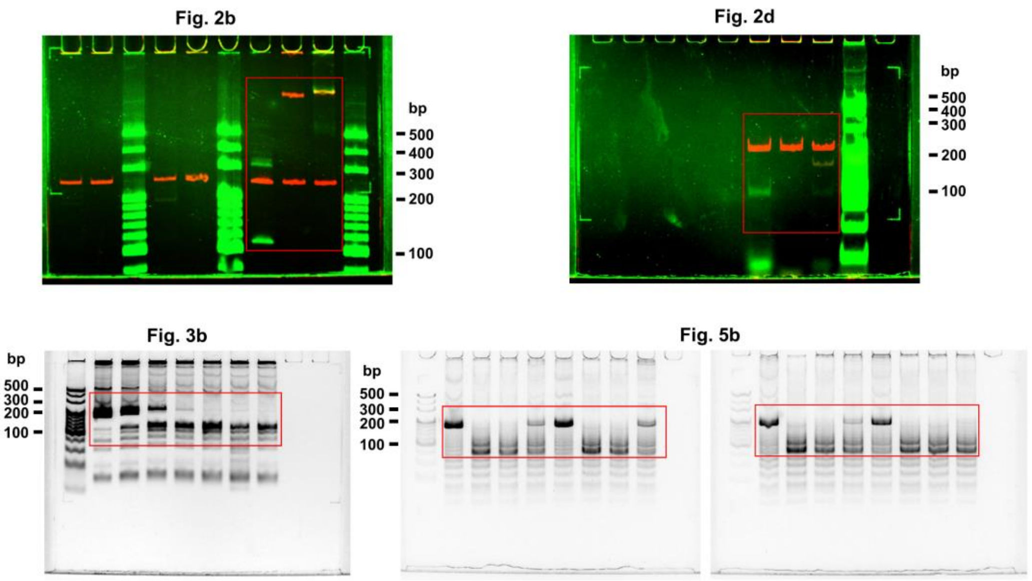

Supplementary Figure 14. Uncropped figures of PAGE gels. Cropped regions are indicated with rectangles as appropriate.

Supplementary Table 1. Sequences of Cas9(H840A)-Cys in plasmid pET28a/Cas9(H840A)-Cys

>Cas9(H840A)-Cys

Labels: Cas9(H840A); 6xHis-Tag; Thrombin Cleavage Site; T7-Tag; H885A mutation of Cas9 HNH catalytic residue; SV40 Nuclear Localization Signal; HA Epitope; stop codon (italic)

ATGGGCAGCAGCCATCATCATCATCATCACAGCAGCGGCCTGGTGCCGCGCGGCAGCCATATGGCTAGCATGA
CTGGTGGACAGCAAATGGGTCGCGGATCCGAATTCGAGCTCCGTCGACAAGCTTGCGGCCGCATGGACAAGAA
GTACAGCATCGGCCTGGACATCGGTACCAACAGCGTGGGCTGGGCCGTGATCACCGACGAGTACAAGGTGCCC
AGCAAGAAGTTCAAGGTGCTGGGCAACACCGACCGCCACAGCATCAAGAAGAACCTGATCGGCGCCCTGCTGT
TCGACAGCGGCGAGACCGCCGAGGCCACCCGCCTGAAGCGCACCGCCCGCCGCCGCTACACCCGCCGCAAGA
ACCGCATCTGCTACCTGCAGGAGATCTTCAGCAACGAGATGGCCAAGGTGGACGACAGCTTCTTCCACCGCCT
GGAGGAGAGCTTCCTGGTGGAGGAGGACAAGAAGCACGAGCGCCACCCCATCTTCGGCAACATCGTGGACGA
GGTGGCCTACCACGAGAAGTACCCCACCATCTACCACCTGCGCAAGAAGCTGGTGGACAGCACCGACAAGGCC
GACCTGCGCCTGATCTACCTGGCCCTGGCCCACATGATCAAGTTCCGCGGCCACTTCCTGATCGAGGGCGACCT
GAACCCCGACAACAGCGACGTGGACAAGCTGTTCATCCAGCTGGTGCAGACCTACAACCAGCTGTTCGAGGAG
AACCCCATCAACGCCAGCGGCGTGGACGCCAAGGCCATCCTGAGCGCCCGCCTGAGCAAGAGCCGCCGCCTGG
AGAACCTGATCGCCCAGCTGCCCGGCGAGAAGAAGAACGGCCTGTTCGGCAACCTGATCGCCCTGAGCCTGGG
CCTGACCCCCAACTTCAAGAGCAACTTCGACCTGGCCGAGGACGCCAAGCTGCAGCTGAGCAAGGACACCTAC
GACGACGACCTGGACAACCTGCTGGCCCAGATCGGCGACCAGTACGCCGACCTGTTCCTGGCCGCCAAGAACC
TGAGCGACGCCATCCTGCTGAGCGACATCCTGCGCGTGAACACCGAGATCACCAAGGCCCCCCTGAGCGCCAG
CATGATCAAGCGCTACGACGAGCACCACCAGGACCTGACCCTGCTGAAGGCCCTGGTGCGCCAGCAGCTGCCC
GAGAAGTACAAGGAGATCTTCTTCGACCAGAGCAAGAACGGCTACGCCGGCTACATCGACGGCGGCGCCAGC
CAGGAGGAGTTCTACAAGTTCATCAAGCCCATCCTGGAGAAGATGGACGGCACCGAGGAGCTGCTGGTGAAGC
TGAACCGCGAGGACCTGCTGCGCAAGCAGCGCACCTTCGACAACGGCAGCATCCCCCACCAGATCCACCTGGG
CGAGCTGCACGCCATCCTGCGCCGCCAGGAGGACTTCTACCCCTTCCTGAAGGACAACCGCGAGAAGATCGAG
AAGATCCTGACCTTCCGCATCCCCTACTACGTGGGCCCCCTGGCCCGCGGCAACAGCCGCTTCGCCTGGATGAC
CCGCAAGAGCGAGGAGACCATCACCCCCTGGAACTTCGAGGAGGTGGTGGACAAGGGCGCCAGCGCCCAGAG
CTTCATCGAGCGCATGACCAACTTCGACAAGAACCTGCCCAACGAGAAGGTGCTGCCCAAGCACAGCCTGCTG
TACGAGTACTTCACCGTGTACAACGAGCTGACCAAGGTGAAGTACGTGACCGAGGGCATGCGCAAGCCCGCCT
TCCTGAGCGGCGAGCAGAAGAAGGCCATCGTGGACCTGCTGTTCAAGACCAACCGCAAGGTGACCGTGAAGC
AGCTGAAGGAGGACTACTTCAAGAAGATCGAGTGCTTCGACAGCGTGGAGATCAGCGGCGTGGAGGACCGCTT
CAACGCCAGCCTGGGCACCTACCACGACCTGCTGAAGATCATCAAGGACAAGGACTTCCTGGACAACGAGGAG
AACGAGGACATCCTGGAGGACATCGTGCTGACCCTGACCCTGTTCGAGGACCGCGAGATGATCGAGGAGCGCC
TGAAGACCTACGCCCACCTGTTCGACGACAAGGTGATGAAGCAGCTGAAGCGCCGCCGCTACACCGGCTGGGG
CCGCCTGAGCCGCAAGCTTATCAACGGCATCCGCGACAAGCAGAGCGGCAAGACCATCCTGGACTTCCTGAAG
AGCGACGGCTTCGCCAACCGCAACTTCATGCAGCTGATCCACGACGACAGCCTGACCTTCAAGGAGGACATCC
AGAAGGCCCAGGTGAGCGGCCAGGGCGACAGCCTGCACGAGCACATCGCCAACCTGGCCGGCAGCCCCGCCA
TCAAGAAGGGCATCCTGCAGACCGTGAAGGTGGTGGACGAGCTGGTGAAGGTGATGGGCCGCCACAAGCCCG
AGAACATCGTGATCGAGATGGCCCGCGAGAACCAGACCACCCAGAAGGGCCAGAAGAACAGCCGCGAGCGCA
TGAAGCGCATCGAGGAGGGCATCAAGGAGCTGGGCAGCCAGATCCTGAAGGAGCACCCCGTGGAGAACACCC
AGCTGCAGAACGAGAAGCTGTACCTGTACTACCTGCAGAACGGCCGCGACATGTACGTGGACCAGGAGCTGGA
CATCAACCGCCTGAGCGACTACGACGTGGACGCCATCGTGCCCCAGAGCTTCCTGAAGGACGACAGCATCGAC
AACAAGGTGCTGACCCGCAGCGACAAGAACCGCGGCAAGAGCGACAACGTGCCCAGCGAGGAGGTGGTGAAG
AAGATGAAGAACTACTGGCGCCAGCTGCTGAACGCCAAGCTGATCACCCAGCGCAAGTTCGACAACCTGACCA
AGGCCGAGCGCGGCGGCCTGAGCGAGCTGGACAAGGCCGGCTTCATCAAGCGCCAGCTGGTGGAGACCCGCC
AGATCACCAAGCACGTGGCCCAGATCCTGGACAGCCGCATGAACACCAAGTACGACGAGAACGACAAGCTGA
TCCGCGAGGTGAAGGTGATCACCCTGAAGAGCAAGCTGGTGAGCGACTTCCGCAAGGACTTCCAGTTCTACAA
GGTGCGCGAGATCAACAACTACCACCACGCCCACGACGCCTACCTGAACGCCGTGGTGGGCACCGCCCTGATC
AAGAAGTACCCCAAGCTGGAGAGCGAGTTCGTGTACGGCGACTACAAGGTGTACGACGTGCGCAAGATGATCG
CCAAGAGCGAGCAGGAGATCGGCAAGGCCACCGCCAAGTACTTCTTCTACAGCAACATCATGAACTTCTTCAA
GACCGAGATCACCCTGGCCAACGGCGAGATCCGCAAGCGCCCCCTGATCGAGACCAACGGCGAGACCGGCGA
GATCGTGTGGGACAAGGGCCGCGACTTCGCCACCGTGCGCAAGGTGCTGAGCATGCCCCAGGTGAACATCGTG
AAGAAGACCGAGGTGCAGACCGGCGGCTTCAGCAAGGAGAGCATCCTGCCCAAGCGCAACAGCGACAAGCTG
ATCGCCCGCAAGAAGGACTGGGACCCCAAGAAGTACGGCGGCTTCGACAGCCCCACCGTGGCCTACAGCGTGC
TGGTGGTGGCCAAGGTGGAGAAGGGCAAGAGCAAGAAGCTGAAGAGCGTGAAGGAGCTGCTGGGCATCACCA
TCATGGAGCGCAGCAGCTTCGAGAAGAACCCCATCGACTTCCTGGAGGCCAAGGGCTACAAGGAGGTGAAGA
AGGACCTGATCATCAAGCTGCCCAAGTACAGCCTGTTCGAGCTGGAGAACGGCCGCAAGCGCATGCTGGCCAG
CGCCGGCGAGCTGCAGAAGGGCAACGAGCTGGCCCTGCCCAGCAAGTACGTGAACTTCCTGTACCTGGCCAGC
CACTACGAGAAGCTGAAGGGCAGCCCCGAGGACAACGAGCAGAAGCAGCTGTTCGTGGAGCAGCACAAGCAC
TACCTGGACGAGATCATCGAGCAGATCAGCGAGTTCAGCAAGCGCGTGATCCTGGCCGACGCCAACCTGGACA
AGGTGCTGAGCGCCTACAACAAGCACCGCGACAAGCCCATCCGCGAGCAGGCCGAGAACATCATCCACCTGTT
CACCCTGACCAACCTGGGCGCCCCCGCCGCCTTCAAGTACTTCGACACCACCATCGACCGCAAGCGCTACACC
AGCACCAAGGAGGTGCTGGACGCCACCCTGATCCACCAGAGCATCACCGGTCTGTACGAGACCCGCATCGACC
TGAGCCAGCTGGGCGGCGACGGCGGCTCCGGACCTCCAAAGAAAAAGAGAAAAGTATACCCCTACGACGTGC
CCGACTACGCCTGT*TAA*

Supplementary Table 2. Sequences of S3C-dCas9 in plasmid pET28a/S3C-dCas9

>S3C-dCas9

Labels: Serine to Cysteine Mutation (S3C); Cas9(H840); 6xHis-Tag; Thrombin Cleavage Site; T7-Tag; D55A (D10A) mutation and H885A (H840A) mutation of Cas9 RuvC and HNH catalytic residue; SV40 Nuclear Localization Signal; HA Epitope; Cysteine to Serine mutation (bold); stop codon (italic)

ATGGGCTGCAGCCATCATCATCATCATCACAGCAGCGGCCTGGTGCCGCGCGGCAGCCATATGGCTAGCAT
GACTGGTGGACAGCAAATGGGTCGCGGATCCGAATTCGAGCTCCGTCGACAAGCT**AGC**GGCCGCATGGAC
AAGAAGTACAGCATCGGCCTGGCCATCGGTACCAACAGCGTGGGCTGGGCCGTGATCACCGACGAGTACAA
GGTGCCCAGCAAGAAGTTCAAGGTGCTGGGCAACACCGACCGCCACAGCATCAAGAAGAACCTGATCGGCG
CCCTGCTGTTCGACAGCGGCGAGACCGCCGAGGCCACCCGCCTGAAGCGCACCGCCCGCCGCCGCTACAC
CCGCCGCAAGAACCGCATC**AGC**TACCTGCAGGAGATCTTCAGCAACGAGATGGCCAAGGTGGACGACAGCT
TCTTCCACCGCCTGGAGGAGAGCTTCCTGGTGGAGGAGGACAAGAAGCACGAGCGCCACCCCATCTTCGGC
AACATCGTGGACGAGGTGGCCTACCACGAGAAGTACCCCACCATCTACCACCTGCGCAAGAAGCTGGTGGA
CAGCACCGACAAGGCCGACCTGCGCCTGATCTACCTGGCCCTGGCCCACATGATCAAGTTCCGCGGCCACT
TCCTGATCGAGGGCGACCTGAACCCCGACAACAGCGACGTGGACAAGCTGTTCATCCAGCTGGTGCAGACC
TACAACCAGCTGTTCGAGGAGAACCCCATCAACGCCAGCGGCGTGGACGCCAAGGCCATCCTGAGCGCCC
GCCTGAGCAAGAGCCGCCGCCTGGAGAACCTGATCGCCCAGCTGCCCGGCGAGAAGAAGAACGGCCTGTT
CGGCAACCTGATCGCCCTGAGCCTGGGCCTGACCCCCAACTTCAAGAGCAACTTCGACCTGGCCGAGGACG
CCAAGCTGCAGCTGAGCAAGGACACCTACGACGACGACCTGGACAACCTGCTGGCCCAGATCGGCGACCA
GTACGCCGACCTGTTCCTGGCCGCCAAGAACCTGAGCGACGCCATCCTGCTGAGCGACATCCTGCGCGTGA
ACACCGAGATCACCAAGGCCCCCCTGAGCGCCAGCATGATCAAGCGCTACGACGAGCACCACCAGGACCTG
ACCCTGCTGAAGGCCCTGGTGCGCCAGCAGCTGCCCGAGAAGTACAAGGAGATCTTCTTCGACCAGAGCAA
GAACGGCTACGCCGGCTACATCGACGGCGGCGCCAGCCAGGAGGAGTTCTACAAGTTCATCAAGCCCATCC
TGGAGAAGATGGACGGCACCGAGGAGCTGCTGGTGAAGCTGAACCGCGAGGACCTGCTGCGCAAGCAGCG
CACCTTCGACAACGGCAGCATCCCCCACCAGATCCACCTGGGCGAGCTGCACGCCATCCTGCGCCGCCAG
GAGGACTTCTACCCCTTCCTGAAGGACAACCGCGAGAAGATCGAGAAGATCCTGACCTTCCGCATCCCCTAC
TACGTGGGCCCCCTGGCCCGCGGCAACAGCCGCTTCGCCTGGATGACCCGCAAGAGCGAGGAGACCATCA
CCCCCTGGAACTTCGAGGAGGTGGTGGACAAGGGCGCCAGCGCCCAGAGCTTCATCGAGCGCATGACCAA
CTTCGACAAGAACCTGCCCAACGAGAAGGTGCTGCCCAAGCACAGCCTGCTGTACGAGTACTTCACCGTGTA
CAACGAGCTGACCAAGGTGAAGTACGTGACCGAGGGCATGCGCAAGCCCGCCTTCCTGAGCGGCGAGCAG
AAGAAGGCCATCGTGGACCTGCTGTTCAAGACCAACCGCAAGGTGACCGTGAAGCAGCTGAAGGAGGACTA
CTTCAAGAAGATCGAG**AGC**TTCGACAGCGTGGAGATCAGCGGCGTGGAGGACCGCTTCAACGCCAGCCTG
GGCACCTACCACGACCTGCTGAAGATCATCAAGGACAAGGACTTCCTGGACAACGAGGAGAACGAGGACAT
CCTGGAGGACATCGTGCTGACCCTGACCCTGTTCGAGGACCGCGAGATGATCGAGGAGCGCCTGAAGACCT
ACGCCCACCTGTTCGACGACAAGGTGATGAAGCAGCTGAAGCGCCGCCGCTACACCGGCTGGGGCCGCCT
GAGCCGCAAGCTTATCAACGGCATCCGCGACAAGCAGAGCGGCAAGACCATCCTGGACTTCCTGAAGAGCG
ACGGCTTCGCCAACCGCAACTTCATGCAGCTGATCCACGACGACAGCCTGACCTTCAAGGAGGACATCCAG
AAGGCCCAGGTGAGCGGCCAGGGCGACAGCCTGCACGAGCACATCGCCAACCTGGCCGGCAGCCCCGCC
ATCAAGAAGGGCATCCTGCAGACCGTGAAGGTGGTGGACGAGCTGGTGAAGGTGATGGGCCGCCACAAGC
CCGAGAACATCGTGATCGAGATGGCCCGCGAGAACCAGACCACCCAGAAGGGCCAGAAGAACAGCCGCGA
GCGCATGAAGCGCATCGAGGAGGGCATCAAGGAGCTGGGCAGCCAGATCCTGAAGGAGCACCCCGTGGAG
AACACCCAGCTGCAGAACGAGAAGCTGTACCTGTACTACCTGCAGAACGGCCGCGACATGTACGTGGACCA
GGAGCTGGACATCAACCGCCTGAGCGACTACGACGTGGACGCCATCGTGCCCCAGAGCTTCCTGAAGGAC
GACAGCATCGACAACAAGGTGCTGACCCGCAGCGACAAGAACCGCGGCAAGAGCGACAACGTGCCCAGCG
AGGAGGTGGTGAAGAAGATGAAGAACTACTGGCGCCAGCTGCTGAACGCCAAGCTGATCACCCAGCGCAAG
TTCGACAACCTGACCAAGGCCGAGCGCGGCGGCCTGAGCGAGCTGGACAAGGCCGGCTTCATCAAGCGCC
AGCTGGTGGAGACCCGCCAGATCACCAAGCACGTGGCCCAGATCCTGGACAGCCGCATGAACACCAAGTAC
GACGAGAACGACAAGCTGATCCGCGAGGTGAAGGTGATCACCCTGAAGAGCAAGCTGGTGAGCGACTTCCG
CAAGGACTTCCAGTTCTACAAGGTGCGCGAGATCAACAACTACCACCACGCCCACGACGCCTACCTGAACG
CCGTGGTGGGCACCGCCCTGATCAAGAAGTACCCCAAGCTGGAGAGCGAGTTCGTGTACGGCGACTACAAG
GTGTACGACGTGCGCAAGATGATCGCCAAGAGCGAGCAGGAGATCGGCAAGGCCACCGCCAAGTACTTCTT
CTACAGCAACATCATGAACTTCTTCAAGACCGAGATCACCCTGGCCAACGGCGAGATCCGCAAGCGCCCCCT
GATCGAGACCAACGGCGAGACCGGCGAGATCGTGTGGGACAAGGGCCGCGACTTCGCCACCGTGCGCAAG
GTGCTGAGCATGCCCCAGGTGAACATCGTGAAGAAGACCGAGGTGCAGACCGGCGGCTTCAGCAAGGAGA
GCATCCTGCCCAAGCGCAACAGCGACAAGCTGATCGCCCGCAAGAAGGACTGGGACCCCAAGAAGTACGG
CGGCTTCGACAGCCCCACCGTGGCCTACAGCGTGCTGGTGGTGGCCAAGGTGGAGAAGGGCAAGAGCAAG
AAGCTGAAGAGCGTGAAGGAGCTGCTGGGCATCACCATCATGGAGCGCAGCAGCTTCGAGAAGAACCCCAT
CGACTTCCTGGAGGCCAAGGGCTACAAGGAGGTGAAGAAGGACCTGATCATCAAGCTGCCCAAGTACAGCC
TGTTCGAGCTGGAGAACGGCCGCAAGCGCATGCTGGCCAGCGCCGGCGAGCTGCAGAAGGGCAACGAGCT
GGCCCTGCCCAGCAAGTACGTGAACTTCCTGTACCTGGCCAGCCACTACGAGAAGCTGAAGGGCAGCCCCG
AGGACAACGAGCAGAAGCAGCTGTTCGTGGAGCAGCACAAGCACTACCTGGACGAGATCATCGAGCAGATC
AGCGAGTTCAGCAAGCGCGTGATCCTGGCCGACGCCAACCTGGACAAGGTGCTGAGCGCCTACAACAAGCA
CCGCGACAAGCCCATCCGCGAGCAGGCCGAGAACATCATCCACCTGTTCACCCTGACCAACCTGGGCGCCC
CCGCCGCCTTCAAGTACTTCGACACCACCATCGACCGCAAGCGCTACACCAGCACCAAGGAGGTGCTGGAC
GCCACCCTGATCCACCAGAGCATCACCGGTCTGTACGAGACCCGCATCGACCTGAGCCAGCTGGGCGGCG
ACGGCGGCTCCGGACCTCCAAAGAAAAAGAGAAAAGTATACCCCTACGACGTGCCCGACTACGCC**AGT***TAA*

Supplementary Table 3. Sequences of oligonucleotides used to study the mechanism of CRISDA

Oligo name	Sequence, 5'-3'	Purpose
$IP_{T1\text{-}DNS}$-Cy3	Cy3-CGTGCTCAGTCTGGG	$IP_{T1\text{-}DNS}$-Cy3 is labeled with Cy3 at 5' end and it is used to bind the exposed region of nontarget strand caused by Cas9 and to initiate strand displacement for CRISDA mechanism study.
$RV3_{Cy5}$	Cy5-CTAGCAAAATAGGCTGTCCC	pGL3-For/Rev is labeled with Cy5 at 5' end, and the primer pair is used to produce pTF1-Cy5 from the pGL3-100-$Target_{WT}$ vector for CRISDA mechanism study.
$GL2_{Cy5}$	Cy5-CTTTATGTTTTTGGCGTCTTCCA	
$IP_{pTF1\text{-}UPS\text{-}47.5}$	TAGATCGGTAAGGATAGCGCTGAGGGCAAGTGCAGGTGCCAGAACATTTCTCTATCGATAGGT	$IP_{pTF1\text{-}UPS\text{-}47.5}$ and $IP_{pTF1\text{-}DNS\text{-}49.7}$ primer pair is used to investigate function of the 3' overhang in IP primers where pTF1 fragment is used as the template. The melting temperatures of the 3' overhang in $IP_{pTF1\text{-}UPS\text{-}47.5}$ and $IP_{pTF1\text{-}DNS\text{-}49.7}$ are 47.5 and 49.7 °C, respectively.
$IP_{pTF1\text{-}DNS\text{-}49.7}$	TAGATCGGTAAGGATAGCGCTGAGGACGTGCTCAGTCTGGGCCTCGAGCCCGGG	
$IP_{pTF1\text{-}UPS\text{-}38.7}$	TAGATCGGTAAGGATAGCGCTGAGGGCAAGTGCAGGTGCCAGAACATTTCTCTATCGA	$IP_{pTF1\text{-}UPS\text{-}38.7}$ and $IP_{pTF1\text{-}DNS\text{-}38}$ primer pair is used to investigate function of the 3' overhang in IP primers where pTF1 fragment is used as the template. The melting temperatures of the 3' overhang in $IP_{pTF1\text{-}UPS\text{-}38.7}$ and $IP_{pTF1\text{-}DNS\text{-}38}$ are 38.7 and 38.0 °C, respectively.
$IP_{pTF1\text{-}DNS\text{-}38}$	TAGATCGGTAAGGATAGCGCTGAGGACGTGCTCAGTCTGGGCCTCGAGCCCG	

Supplementary Table 4. Sequences of DNA oligonucleotides for generating CRISDA templates in study

Oligo name	Sequence, 5'-3'	Purpose
pTF1-For	TACGGGAGGTACTTGGAGC	pTF1-For/Rev primer pair is used to amplify the partial sequence of the pGL3-100-$Target_{WT}$ vector and mutated plasmids to produce the pTF1 fragment and mutated fragments for CRISDA detection.
pTF1-Rev	TATGCAGTTGCTCTCCAGCG	
hTF1-For	ACTGCAGGTGCAAAGGCCCG	hTF1-For/ Rev primer pair is used to amplify the partial sequence from the human genome to produce the hTF1 fragment for CRISDA detection.
hTF1-Rev	TGAGGCTGGCCCCTTCCAGG	
hTF2-For	TAGTCCTTGGCTGTTCTGTGAT	hTF2-For/ Rev primer pair is used to amplify the partial sequence from the human genome to produce hTF2 fragment for CRISDA detection.
hTF2-Rev	TTGTATGTTGTCCTGCCTGTTT	
gTF1-For	AGCGAATTACAACTCAACCA	gTF1-For/ Rev primer pair is used to amplify the partial sequence from the MON87705 genome to produce the gTF1 fragment for CRISDA detection.
gTF1-Rev	TTTCAAAGATGCCCACTAAC	

Supplementary Table 5. Sequences of DNA oligonucleotides for constructing different pDR274-sgRNA expression plasmids

Oligo name	Sequence, 5'-3'	Purpose
pTF1-DNS-sgFor	TAGGGGCCCAGACTGAGCACGTGA	Annealed products of the two oligos are ligated with BsaI digested pDR274 to construct the derived plasmid for *in vitro* transcription of sgRNA $sg_{pTF1\text{-}DNS}$.
pTF1-DNS-sgRev	AAACTCACGTGCTCAGTCTGGGCC	
pTF1-UPS-sgFor	TAGGTCTGGCACCTGCACTTGCAC	Annealed products of the two oligos are ligated with BsaI digested pDR274 to construct the derived plasmid for *in vitro* transcription of sgRNA $sg_{pTF1\text{-}UPS}$.
pTF1-UPS-sgRev	AAACGTGCAAGTGCAGGTGCCAGA	
hTF1-DNS-sgFor1	TAGGCTTGTAGCTACGCCTGTGAT	Annealed products of the two oligos are ligated with BsaI digested pDR274 to construct the derived plasmid for *in vitro* transcription of sgRNA $sg_{hTF1\text{-}DNS1}$.
hTF1-DNS-sgRev1	AAACATCACAGGCGTAGCTACAAG	
hTF1-UPS-sgFor1	TAGGTTGCAACTGGCCTCAACCTT	Annealed products of the two oligos were ligated with BsaI digested pDR274 to construct derived plasmid for further study of generating $sg_{hTF1\text{-}UPS1}$.
hTF1-UPS-sgRev1	AAACAAGGTTGAGGCCAGTTGCAA	
hTF1-DNS-sgFor2	TAGGGGCCCAGACTGAGCACGTGA	Annealed products of the two oligos are ligated with BsaI digested pDR274 to construct the derived plasmid for *in vitro* transcription of sgRNA $sg_{hTF1\text{-}DNS2}$.
hTF1-DNS-sgRev2	AAACTCACGTGCTCAGTCTGGGCC	
hTF1-UPS-sgFor2	TAGGCCCTTGCTTAAAACTCTCCA	Annealed products of the two oligos are ligated with BsaI digested pDR274 to construct the derived plasmid for *in vitro* transcription of sgRNA $sg_{hTF1\text{-}UPS2}$.
hTF1-UPS-sgRev2	AAACTGGAGAGTTTTAAGCAAGGG	
hTF2-DNS-sgFor	TAGGAACTACCCAGTATTTGTTTC	Annealed products of the two oligos are ligated with BsaI digested pDR274 to construct the derived plasmid for *in vitro* transcription of sgRNA $sg_{hTF2\text{-}DNS}$.
hTF2-DNS-sgRev	AAACGAAACAAATACTGGGTAGTT	
hTF2-UPS-sgFor	TAGGCACAGTTTTATTCTTCGCTA	Annealed products of the two oligos are ligated with BsaI digested pDR274 to construct the derived plasmid for *in vitro* transcription of sgRNA $sg_{hTF2\text{-}UPS}$.
hTF2-UPS-sgRev	AAACTAGCGAAGAATAAAACTGTG	
gTF1-DNS-sgFor	TAGGTACGATCCGTCGTATTTATA	Annealed products of the two oligos are ligated with BsaI digested pDR274 to construct the derived plasmid for *in vitro* transcription of sgRNA $sg_{gTF1\text{-}DNS}$.
gTF1-DNS-sgRev	AAACTATAAATACGACGGATCGTA	
gTF1-UPS-sgFor	TAGGTTAGTGATTTCTCCCTTTAT	Annealed products of the two oligos are ligated with BsaI digested pDR274 to construct the derived plasmid for *in vitro* transcription of sgRNA $sg_{gTF1\text{-}UPS}$.
gTF1-UPS-sgRev	AAACATAAAGGGAGAAATCACTAA	

Supplementary Table 6. Sequences of initiating primer (IP) pairs to trigger exponential amplification in CRISDA reactions

Oligo name	Sequence, 5'-3'	Purpose
$IP_{pTF1\text{-}UPS}$	TAGATCGGTAAGGATAGCGCTGAGGGCAAGTGCAGGTGCCAGAACATTTCTCTATCGATAGGTACC	$IP_{pTF1\text{-}UPS/DNS}$ primer pair is used for amplification in the CRISDA reaction where pTF1 fragment and its mutated fragments are used as templates.
$IP_{pTF1\text{-}DNS}$	TAGATCGGTAAGGATAGCGCTGAGGACGTGCTCAGTCTGGGCCTCGAGCCCGGGCTAG	
$IP_{hTF1\text{-}UPS1}$	TAGATCGGTAAGGATAGCGCTGAGGGGTTGAGGCCAGTTGCAAAGACAATTGACATGTTACATTTTG	$IP_{hTF1\text{-}UPS1/DNS}$ primer pair is used for amplification in the CRISDA reaction where hTF1 fragment is used as the template.
$IP_{hTF1\text{-}DNS1}$	TAGATCGGTAAGGATAGCGCTGAGGCACAGGCGTAGCTACAAGATTAGTTTTGAGACTCTCATTCTA	
$IP_{hTF1\text{-}UPS2}$	TAGATCGGTAAGGATAGCGCTGAGGGAGAGTTTTAAGCAAGGGCTGATGTGGGCTGCCTAGA	$IP_{hTF1\text{-}UPS2/DNS2}$ primer pair is used for amplification in the CRISDA reaction where hTF1 fragment is used as the template.
$IP_{hTF1\text{-}DNS2}$	TAGATCGGTAAGGATAGCGCTGAGGACGTGCTCAGTCTGGGCCCCAAGGATTGACCCAGGC	
$IP_{hTF2\text{-}UPS}$	TAGATCGGTAAGGATAGCGCTGAGGACGAAGAATAAAACTGTGGGACTGACCCCCACCCAT	$IP_{hTF2\text{-}UPS/DNS}$ primer pair is used for amplification in the CRISDA reaction where hTF2 fragment is used as the template.
$IP_{hTF2\text{-}DNS}$	TAGATCGGTAAGGATAGCGCTGAGGAACAAATACTGGGTAGTTATTATTTTGCTTAAGTGAAAAACA	
$IP_{gTF1\text{-}UPS}$	TAGATCGGTAAGGATAGCGCTGAGGAAAGGGAGAAATCACTAAGTTTGTGGTTCAGTCCGG	$IP_{gTF1\text{-}UPS/DNS}$ primer pair is used for amplification in the CRISDA reaction where gTF1 fragment is used as the template.
$IP_{gTF1\text{-}DNS}$	TAGATCGGTAAGGATAGCGCTGAGGAAATACGACGGATCGTAATTTGTCGTTTTATCAAAATGTA	

Supplementary Table 7. Sequences of PCR primer pair to compare the performance between CRISDA and PCR

Oligo name	Sequence, 5’-3’	Purpose
GMO-For	CCAATAAAGGGAGAAATCACTAA	GMO-For/Rev primer pair is used in PCR reactions to amplify fragments from gTF1 fragment and MON87705 genome, in order to compare the performance between CRISDA and PCR.
GMO-Rev	CCTATAAATACGACGGATCGTAA	

Supplementary Table 8. Sequences of oligonucleotides for generating pGL3-100-Targe$_{WT}$ vector and its mutants

Oligo name	Sequence, 5'-3'	Purpose
pGL3-100-For	GTCTGCCTAAAGGTGTCGCT	A Luciferase coding region is amplified from pGL-3 plasmid by the two oligos and inserted in SacI and NheI digested pGL-3 Vector to generate the pGL3-100 vector.
pGL3-100-Rev	CGCAGTATCCGGAATGATTTGA	
pTF1$_{WT}$-For	TCGAGGCCCAGACTGAGCACGTGATGG	Annealed products of the two oligos are ligated with the pGL3-100 vector digested by XhoI and HindIII to construct mutated plasmid at PAM sequence (pGL3-100-Target$_{WT}$), used to produce templates for testing specificity of CRISDA.
pTF1$_{WT}$-For	AGCTCCATCACGTGCTCAGTCTGGGCC	
pTF1$_{MP}$-For	TCGAGGCCCAGACTGAGCACGTGATTG	Annealed products of the two oligos are ligated with the pGL3-100 vector digested by XhoI and HindIII to construct mutated plasmid at PAM sequence (pGL3-100-Target$_{MP}$), used to produce templates for testing specificity of CRISDA.
pTF1$_{MP}$-Rev	AGCTCAATCACGTGCTCAGTCTGGGCC	
pTF1$_{M1}$-For	TCGAGGCCCAGACTGAGCACGTGCTGG	Annealed products of the two oligos are ligated with the pGL3-100 vector digested by XhoI and HindIII to construct mutated plasmid at the first nucleotide close to PAM end (pGL3-100-Target$_{M1}$), used to produce templates for testing specificity of CRISDA.
pTF1$_{M1}$-Rev	AGCTCCAGCACGTGCTCAGTCTGGGCC	
pTF1$_{M2}$-For	TCGAGGCCCAGACTGAGCACGTAATGG	Annealed products of the two oligos are ligated with the pGL3-100 vector digested by XhoI and HindIII to construct mutated plasmid at the second nucleotide close to PAM end (pGL3-100-Target$_{M2}$), used to produce templates for testing specificity of CRISDA.
pTF1$_{M2}$-Rev	AGCTCCATTACGTGCTCAGTCTGGGCC	
pTF1$_{M3}$-For	TCGAGGCCCAGACTGAGCACGCGATGG	Annealed products of the two oligos are ligated with the pGL3-100 vector digested by XhoI and HindIII to construct mutated plasmid at the third nucleotide close to PAM end (pGL3-100-Target$_{M3}$), used to produce templates for testing specificity of CRISDA.
pTF1$_{M3}$-Rev	AGCTCCATCGCGTGCTCAGTCTGGGCC	
pTF1$_{M4}$-For	TCGAGGCCCAGACTGAGCACATGATGG	Annealed products of the two oligos are ligated with the pGL3-100 vector digested by XhoI and HindIII to construct mutated plasmid at the forth nucleotide close to PAM end (pGL3-100-Target$_{M4}$), used to produce templates for testing specificity of CRISDA.
pTF1$_{M4}$-Rev	AGCTCCATCATGTGCTCAGTCTGGGCC	
pTF1$_{M5}$-For	TCGAGGCCCAGACTGAGCAGGTGATGG	Annealed products of the two oligos are ligated with the pGL3-100 vector digested by XhoI and HindIII to construct mutated plasmid at the fifth nucleotide close to PAM end (pGL3-100-Target$_{M5}$), used for producing templates testing specificity of CRISDA.
pTF1$_{M5}$-Rev	AGCTCCATCACCTGCTCAGTCTGGGCC	

Supplementary Table 9. PNAs used in the study for endpoint measurements

PNA name	Sequence, N to C terminal	Purpose
PNA_{pTF1}-Cy5	Cy5-GCCTAAAGGTGTCGCTCTG	PNA_{pTF1}-biotin/Cy5 are used to invade CRISDA products generated by the $IP_{pTF1-UPS/DNS}$ primer pair from pTF1 fragment and its mutated fragments for separating specific product from non-specific products
PNA_{pTF1}-biotin	TGGCAATCAAATCATTCCG-biotin	
$PNA_{hTF1-UPS1/DNS1}$-Cy5	Cy5-CTTGACGGCTTTCTTGT	$PNA_{hTF1-UPS1/DNS1}$-biotin/Cy5 are used to invade CRISDA products generated by $IP_{hTF1-UPS1/DNS1}$ primer pair from hTF1 fragment for separating specific product from non-specific products
$PNA_{hTF1-UPS1/DNS1}$-biotin	CAGTTTTGGAGGATGTA-biotin	
$PNA_{hTF1-UPS2/DNS2}$-Cy5	Cy5-TATTTCTGCTGCAAGTAAG	$PNA_{hTF1-UPS2/DNS2}$-biotin/Cy5 are used to invade CRISDA products generated by $IP_{hTF1-UPS2/DNS2}$ primer pair from hTF1 fragment for separating specific product from non-specific products
$PNA_{hTF1-UPS2/DNS2}$-biotin	TTCTGCTTCTCCAGCCCTG-biotin	
PNA_{hTF2}-Cy5	Cy5-ACTGGGTCTTCAGCTTTCA	PNA_{hTF2}-biotin/Cy5 are used to invade CRISDA products generated by $IP_{pTF2-UPS/DNS}$ primer pair from hTF2 fragment for separating specific product from non-specific products
PNA_{hTF2}-biotin	GTTCAGCCGGTGGTCTTT-biotin	
PNA_{gTF1}-Cy5	Cy5-GAGTATGATGGTCAATATGG	PNA_{gTF1}-biotin/Cy5 are used to invade CRISDA products generated by $IP_{gTF1-UPS/DNS}$ primer pair from gTF1 fragment for separating specific product from non-specific products
PNA_{gTF1}-biotin	TGTAGATGTCCGCAGCGTTAT-biotin	

Supplementary Table 10. Summary of sgRNAs applied to detect regions in the human genome in this study

sgRNA Name	Sequence in the guide region (5' ~ 3')	Distance between sg_{UPS} and sg_{DNS}	sgRNA performance analyzed by CRISPR Design (V1)*	
			Score	Number of potential off-target sites in the human genome
$sg_{hTF1\text{-}DNS1}$	CUUGUAGCUACGCCUGUGAU	169 bp	85	60
$sg_{hTF1\text{-}UPS1}$	UUGCAACUGGCCUCAACCUU		77	148
$sg_{hTF1\text{-}DNS2}$	GGCCCAGACUGAGCACGUGA	203 bp	65	275
$sg_{hTF1\text{-}UPS2}$	CCCUUGCUUAAAACUCUCCA		55	312
$sg_{hTF2\text{-}DNS}$	AACUACCCAGUAUUUGUUUC	194 bp	63	243
$sg_{hTF2\text{-}UPS}$	CACAGUUUUAUUCUUCGCUA		74	205

* sequences are analyzed by an online tool, CRISPR Design (V1, Zhang's Lab, MIT, 2013), at: http://crispr.mit.edu/.

Supplementary Table 11. The GC-content and melting temperature of the middle region in IP primers used in this study.

IP Primer Name	The middle hybridization region complementary to the exposed nontarget strand	
	GC content	*T*m
$IP_{pTF1\text{-}UPS}$	62.5%	54 ºC
$IP_{pTF1\text{-}DNS}$	68.8%	56.2 ºC
$IP_{hTF1\text{-}UPS1}$	56.2 %	52.8 ºC
$IP_{hTF1\text{-}DNS1}$	50.0 %	50.0 ºC
$IP_{hTF1\text{-}UPS2}$	37.5 %	42.6 ºC
$IP_{hTF1\text{-}DNS2}$	68.8 %	56.2 ºC
$IP_{hTF2\text{-}UPS}$	31.2 %	39.8 ºC
$IP_{hTF2\text{-}DNS}$	37.5 %	42.9 ºC
$IP_{gTF1\text{-}UPS}$	37.5 %	42.6 ºC
$IP_{gTF1\text{-}DNS}$	43.8 %	46.2 ºC
